# Seasonal Dynamics of Non-Biting Midges (Diptera: Chironomidae) and Relevant Environmental Factors

**DOI:** 10.3390/insects15120921

**Published:** 2024-11-25

**Authors:** Teng Lei, Jingjing Gu, Mengyao Zhao, Yuqiu Chen, Chao Song, Xin Qi

**Affiliations:** 1 Zhejiang Provincial Key Laboratory of Plant Evolutionary Ecology and Conservation, School of Life Sciences, Taizhou University, Taizhou 318000, China; leiteng@tzc.edu.cn (T.L.);; 2 Managing Committee of Xianju National Park, Xianju 317300, China; gujj0112@163.com; 3 Taizhou Pollution Control Technology Center Co., Ltd., Taizhou 318000, China; baitu119110@126.com

**Keywords:** Chironomidae, non-biting midge, seasonal dynamics, environmental conditions

## Abstract

The family Chironomidae is speciose and is present in almost all freshwater habitats. Adult non-biting midges emerge from waterbodies and swarm in high numbers, occasionally disrupting people’s outdoor activities. In order to understand the seasonal dynamics of species composition, a continuous observation of non-biting midge diversity was performed. Adult non-biting midges were collected using light traps from the autumn of 2022 to the summer of 2023 in an urban wetland park. Species were identified based on morphological characteristics and DNA barcodes. Alpha diversity was evaluated using Margalef, Pielou, and Shannon–Wiener indexes. Beta diversity was evaluated using unconstrained NMDS analysis and constrained CCA. The impacts of environmental factors, including barometric pressure, temperature, relative humidity, and wind speed, on the variation in species composition were estimated in the constrained analyses. A total of 42 species were identified, with 29 species belonging to Chironominae, 9 species belonging to Orthocladiinae, and 4 species belonging to Tanypodinae. The species composition varied across different seasons. Summer sites and autumn sites shared the highest similarity in diversity, and spring sites presented the lowest diversity. The variation was significantly correlated with environmental conditions. The results showed that seasonality is a factor influencing the diversity of adult non-biting midges.

## Simple Summary

Non-biting midges cause a nuisance by swarming near areas where human activities are conducted. Numerous studies have focused on the taxonomic and functional diversity of non-biting midges, while their seasonal population dynamics are less studied. In order to understand the activity patterns of adult non-biting midges, we observed their species diversity continuously over different seasons in an urban wetland park. The species composition of non-biting midges differed significantly in different seasons. Environmental factors such as barometric pressure, temperature, relative humidity, and wind speed were recorded during sampling, and the species variation was significantly correlated with these factors. The results extend our knowledge of the seasonal dynamics of non-biting midge populations and provide a basis for developing strategies to mitigate the hazards of this assemblage.

## 1. Introduction

The family of Chironomidae (Diptera: Nematocera) is called “non-biting midges”. Non-biting midges are a considerable nuisance in urban areas when present in high numbers. They swarm at dawn or dusk near buildings and are attracted by artificial light sources, disrupting people’s normal outdoor activities [1]. They are also possible windborne carriers of *Vibrio cholerae* non-O1 and non-O39, which are associated with major epidemics [2]. Several species in the genera *Chironomus*, *Tokunagayusurika*, and *Polypedilum* are potent allergens that elicit allergic reactions in humans [3,4,5]. In order to avoid the negative effects of non-biting midges, we need to learn about their diversity and relevant influencing factors.

The adult stage is the only terrestrial stage for most Chironomidae species. The eggs, larvae, and pupae live a benthic or aquatic life [6]. Previous studies have mostly focused on the diversity and distribution of benthic macroinvertebrates [7,8,9] and the relevant environmental factors explaining their distribution [10,11]. The diversity of the larvae varies with different factors, such as water temperature, salinity, dissolved oxygen, and nutrient content [10]. Seasonality is also a strong factor in predicting the variation in larval diversity. The species diversity is most similar in summer and autumn and lowest in winter [11]. Few studies have focused on unraveling and explaining the diversity of adults. The dispersal of adults in agricultural landscapes is influenced by the distance to waterbodies, the quality of the hedge, the density of the hedgerow, and landscape openness [12]. In this work, we aim to explore the influence of seasonality on the diversity of adults.

Urban parks built on wetlands, where residents are annoyed by non-biting midges most of the year, are ideal for long-term observations of non-biting midge diversity. The waterbodies and sediments of wetlands provide larvae with shelter and sufficient nutrients, leading to the increased abundance of larvae and subsequent adults. Although urban water bodies may have a high load of organic matter and metals [13], non-biting midges are tolerant to these pollutants [14]. Non-biting midges are ubiquitous and abundant in such waterbodies. They emerge from the waterbodies and swarm in the nearby areas. Seasonal variation in the larvae of non-biting midges of wetlands has been observed [15]; this variation might also be observed in adults.

The Jinyang Wetland Park is built on a wetland. It is located in the center of Taizhou City, Zhejiang Province, China, with the urban Yongning River flowing on its eastern side. We studied non-biting midges in this area, unraveling their biodiversity and relevant environmental factors, aiming to understand their behavioral patterns.

## 2. Materials and Methods

### 2.1. Sampling

Three sampling sites were determined within the Jinyang Wetland Park (Site 1, 28.660565° N, 121.390422° E; Site 2, 28.659947° N, 121.388553° E; Site 3, 28.660181° N, 121.387404° E), all of which were located on the lawn between the waterbody and the trail. Sampling was conducted from the autumn of 2022 to the summer of 2023, with three replicates per season at each sampling site. Environmental conditions, such as barometric pressure, temperature, relative humidity, and wind speed, were recorded at the time of sampling (Table 1). The environmental variables were compared between seasons using one-way ANOVA after the Shapiro–Wilk normality test and Bartlett test of homogeneity of variances performed using R-package ‘multcomp’. Sampling was conducted using light traps. We sampled for an hour immediately after sunset. When sampling, the non-biting midges were attracted to eight 1.12 m^2^ white screens surrounding a 400 W mercury lamp. The non-biting midges on the screens were all collected by aspiration with a suction sampler and sorted in 75% alcohol until sorting.

### 2.2. Species Identification

Species identification was conducted based on morphological characteristics and DNA barcodes of cytochrome *c* oxidase subunit I (*COI*). In brief, the wings, legs, and hypopygium of each specimen were inspected under a microscope, and the morphological characteristics were compared to taxa keys [16,17,18,19,20,21,22,23,24,25,26,27,28,29,30,31,32,33,34,35] to identify species. The specimens sharing the same morphological characteristics were regarded as the same species, and their scientific names were verified according to the taxa keys. Samples that were not completely matched to the taxa keys were considered as putative species. To further confirm the species identification results, the *COI* genes from about 5 percent of the specimens of every identified or putative species were amplified using the universal primers LCO1490 and HCO2198 [36] and sequenced. The *COI* sequences were used to delineate species using the Generalized Mixed Yule Coalescent (GMYC) method [37,38]. Briefly, a Yule model and a constant clock were used to build an ultrametric tree in BEAST v1.10.4. The model of molecular evolution was set as HKY, and the mcmc chain length was set as 60 million until all values of Estimated Sample Sizes (ESSs) reached 200. The resultant tree was used for species delimitation using the R-package ‘splits’ v1.0.20. Then, the *COI* sequences were submitted to the Barcode of Life Data System (BOLD) at https://www.boldsystems.org/index.php (accessed on 1 August 2024) for species identification. The sequences that failed to be identified by BOLD were then submitted to the National Center for Biotechnology Information (NCBI) to run blast against its standard databases (https://www.ncbi.nlm.nih.gov/, accessed on 1 August 2024). All sequences were uploaded to the NCBI to obtain GenBank accession numbers. The number of specimens per species of each sampling event was recorded and used for diversity analysis. The top 15 genera and top 15 species were individually visualized using R-packages ‘vegan’ v2.6.4 and ‘reshape2’ v1.4.4.

### 2.3. Alpha Diversity Analyses

Based on the number of specimens per species of each sampling event, the Margalef, Pielou, and Shannon–Wiener indexes were calculated to estimate alpha diversity. The Margalef [39] index was used to evaluate the richness of species. The Pielou [40] index was used to evaluate the evenness of species. The Shannon–Wiener [41] index was used to evaluate the diversity of species. The number of observed species *S* were calculated by the ‘estimateR’ function in R-package ‘vegan’. The Margalef (*d*) index was calculated using the following equation, *d* = (*S* − 1)/ln *N*, where *N* indicates the total number of specimens. The Shannon–Wiener (*H*′) index was calculated by the ‘diversity’ function in R-package ‘vegan’. The Pielou (*J*) index was calculated using the following equation: *J* = *H*′/ln *S*. The samples were divided into three groups by season. The indexes between the groups were analyzed using one-way ANOVA after the Shapiro–Wilk normality test and Bartlett test of homogeneity of variances.

### 2.4. Beta Diversity Analyses

Beta diversity was analyzed using unconstrained non-metric multidimensional scaling (NMDS) and constrained canonical correspondence analysis (CCA). NMDS is a distance-based analysis. The Bray–Curtis distance is more suitable for species with high abundance and was used for NMDS in this study. The NMDS analysis was conducted using the ‘metaMDS’ function of R-package ‘vegan’. Environmental factors were applied in the constrained analyses of CCA. Prior to the constrained analysis, detrended correspondence analysis (DCA) was performed to estimate the fitness of the linear model and unimodal model for the data in this study. The DCA was performed using the ‘decorana’ function. The resultant axis length of DCA1 was 3.8072, between 3.0 and 4.0. Thus, the linear model and unimodal model were both suitable for the data. We chose the unimodal model because more variation was explained in our results. In the unimodal model analyses, raw data were used to conduct correspondence analysis (CA) with the ‘cca’ function, followed by incorporating environmental factors to perform CCA. The regression analysis between chironomid diversity and environmental factors was performed using the ‘envfit’ function.

## 3. Results

### 3.1. Composition of Non-Biting Midges

From the autumn of 2022 to the summer of 2023, 2640 non-biting midges were collected using light traps. These non-biting midges were identified as belonging to 39 species and 3 putative species according to morphological characteristics. The *COI* genes were amplified from 123 specimens across the 42 (putative) species. The sequences were deposited in the NCBI under accession numbers PQ340971–PQ341093 (Table S1).The results of GMYC species delimitation supported the finding that the specimens included 42 species (LR test: *p* < 0.001). The *COI* sequences from 40 species were matched to BOLD systems with similarities of over 98.08%. The sequences from the other two species were matched to NCBI datasets with similarities of over 99.37% (Table 2). These results strongly support the results of species identification. Among the species, one from *Polypedilum*, one from *Smittia*, and one from *Procladius* were not identified with specific scientific names. However, similar *COI* sequences had been deposited in BOLD systems prior to this work. Thus, they were treated as species in our study. Taken together, 42 non-biting midge species were found at Jinyang Wetland Park, belonging to 20 genera and three subfamilies, i.e., Chironominae, Orthocladiinae, and Tanypodinae.

**Figure 1:**
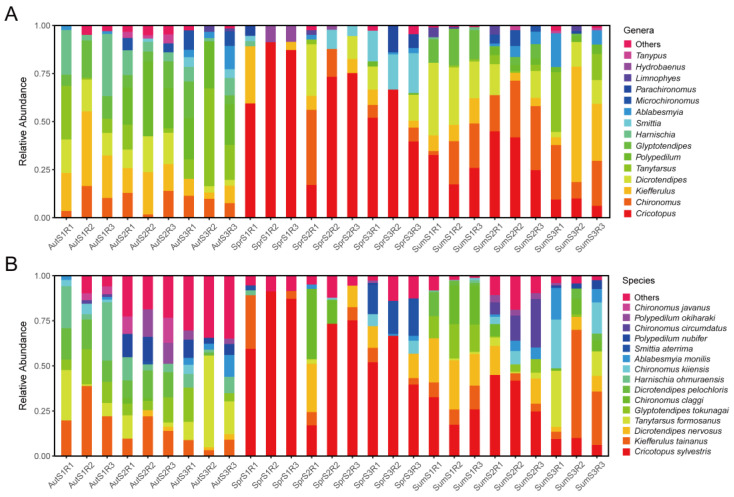
Relative abundance of non-biting midges. (**A**) Relative abundance of non-biting midges at genus level. Top 15 abundant genera are displayed one by one, and the rest are grouped into ‘Others’. (**B**) Relative abundance of non-biting midges at species level. Top 15 abundant species are displayed one by one, and the rest are grouped into ‘Others’.

In the autumn of 2022, 596 specimens were collected. They were identified as belonging to 31 species from 15 genera. The most abundant genera were *Kiefferulus*, *Tanytarsus,* and *Polypedilum*. The most abundant species were *Kiefferulus tainanus*, *Tanytarsus formosanus,* and *Harnischia ohmuraensis*. In the spring of 2023, 812 specimens were collected. They were identified as belonging to 19 species from 15 genera. The most abundant genera were *Cricotopus*, *Smittia,* and *Dicrotendipes*. The most abundant species were *Cricotopus sylvestris*, *Smittia aterrima,* and *Dicrotendipes nervosus*. In the summer of 2023, 1232 specimens were collected. They were identified as belonging to 25 species from 16 genera. The most abundant genera were *Cricotopus*, *Chironomus,* and *Dicrotendipes*. The most abundant species were *Cricotopus sylvestris*, *Dicrotendipes nervosus,* and *Kiefferulus tainanus*. *Endochironomus* was only collected in autumn. *Benthalia* and *Hydrobaenus* were only collected in spring. No genus was only collected in summer. *Chironomus*, *Dicrotendipes*, *Harnischia*, *Kiefferulus*, *Polypedilum*, *Tanytarsus*, *Limnophyes*, *Ablabesmyia,* and *Procladius* were found in all the seasons (Figure 1; Table S2).

### 3.2. Alpha Diversity

**Figure 2:**
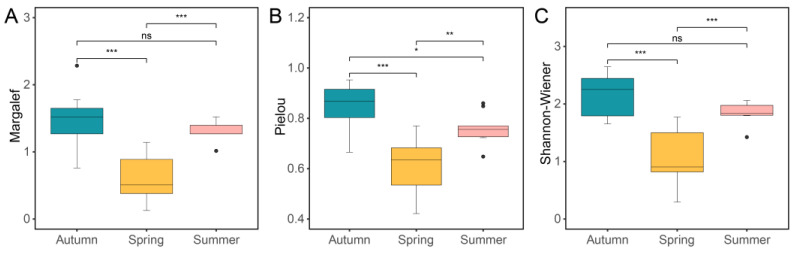
Diagrams of alpha diversity indexes. (**A**) Margalef. (**B**) Pielou. (**C**) Shannon–Wiener. Samples are grouped by season. The indexes between groups are analyzed using one-way ANOVA after Shapiro–Wilk normality test and Bartlett test of homogeneity of variances. ns, non-significance; *, *p* < 0.05; **, *p* < 0.01; ***, *p* < 0.001.

The Margalef index was used to estimate the abundance of non-biting midge species across the three seasons. There was no significant difference in this index between autumn and summer. The values of spring (Margalef 0.58 ± 0.12) were significantly lower than those of autumn (Margalef 1.38 ± 0.11) and summer (Margalef 1.35 ± 0.04), implying the lowest species abundance in spring (Figure 2A). The Pielou index was used to estimate the evenness of species. The values of the three seasons showed significant difference between the pairings. The evenness was the highest in autumn (Pielou 0.85 ± 0.03), medium in summer (Pielou 0.75 ± 0.01), and the lowest in spring (Pielou 0.61 ± 0.04) (Figure 2B). The lowest evenness in spring resulted from the dominant species *Cricotopus sylvestris*, which accounted for 67.73% of the spring specimens. The Shannon–Wiener index was used to estimate the diversity of species. Similarly to the abundance of species, there were no significant differences in the Shannon–Wiener index between autumn and summer. The values of spring (Shannon–Wiener 1.02 ± 0.17) were significantly lower than those of autumn (Shannon–Wiener 2.14 ± 0.13) and summer (Shannon–Wiener 1.90 ± 0.04), implying the lowest species diversity in spring (Figure 2C).

### 3.3. Beta Diversity

**Figure 3:**
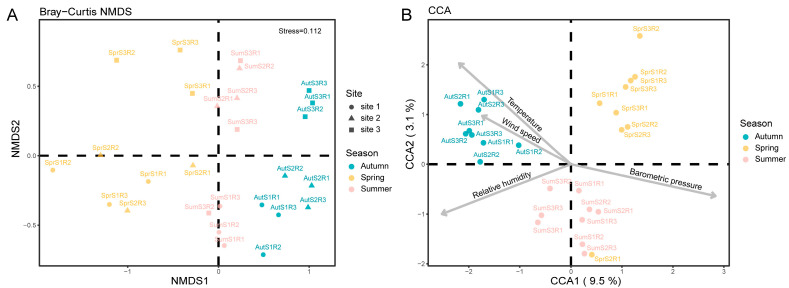
Diagrams of beta diversity. (**A**) NMDS analysis based on Bray–Curtis distance. (**B**) Raw-data-based CCA using unimodal model. Seasons are distinguished by plot colors. Sampling sites are distinguished by plot shapes.

NMDS is an unconstrained analysis. In the Bray–Curtis-distance-based NMDS analysis, the stress value was 0.112, implying the reliable results of NMDS. The spring plots were obviously separated from the autumn plots. The summer plots were close to both the spring plots and the autumn plots (Figure 3A). The plots from the same sampling site were not grouped together, implying that the sampling sites had little effect on the non-biting midge diversity in this study. Thus, we mainly focused on the effects of seasons on the diversity. Based on the results of the NMDS analysis, we concluded that seasons had a significant effect on non-biting midge diversity, and the diversity was obviously different in spring and autumn.

Environmental variables varied across seasons. The average barometric pressure was significantly higher in autumn (1012.96 ± 0.45 hPa) and spring (1011.88 ± 0.47 hPa) than in summer (1002.06 ± 0.74 hPa) (*p* < 0.001), while autumn and spring presented similar barometric pressure (*p* = 0.117). The average temperature was the lowest in spring (17.05 ± 0.26 °C), medium in autumn (20.29 ± 0.58 °C), and highest in summer (29.49 ± 0.60 °C). The differences in average temperature were all significant with *p* < 0.001. The average relative humidity was the lowest in autumn (71.09 ± 0.25%), medium in spring (77.35 ± 0.43%), and highest in summer (83.33 ± 2.13%). The differences in relative humidity were all significant with *p* < 0.05. The average wind speed was significantly (*p* < 0.05) lower in autumn (2.58 ± 0.34 m/s) and spring (2.28 ± 0.18 m/s) than in summer (3.61 ± 0.23 m/s). Autumn and spring presented similar wind speed (*p* = 0.455). The species’ responses to environmental factors were analyzed using unimodal-model-based CCA. In CCA, the constrained factors accounted for 27.41% of inertia. The adjusted r^2^ was 0.1451, and the adjusted components accounted for 9.5% (CCA1) and 3.1% (CCA2). The plots of different seasons were separated from each other (Figure 3B). Environmental factors, i.e., barometric pressure (*p* < 0.001), temperature (*p* < 0.001), relative humidity (*p* < 0.001), and wind speed (*p* < 0.05), were all significantly correlated with chironomid community variation.

## 4. Discussion

Our study provides a list of 42 species of adult non-biting midges found at the urban Jinyang Wetland Park. Previous publications mostly focused on the diversity of Chironomidae larvae and pupae because of their importance for determining variations in the ecological conditions of aquatic habitats [42,43]. A few studies investigated the diversity of adults to find potential biological agents of aquatic weeds [44] or to supplement the results of a biodiversity survey based on larvae [30]. We investigated the diversity of adult non-biting midges because they cause disruptions to human activities in urban parks. Our results showed that 29 out of 42 species belonged to the subfamily Chironominae. Only 13 species belonged to Orthocladiinae and Tanypodinae. In a biodiversity survey performed in an urban river in southeastern Brazil, genera of Chironominae were found to be associated with more organically polluted sampling sites, while Orthocladiinae and Tanypodinae were found to be associated with the sites upstream of the urban area [42]. Compared with rural waterbodies, urban waterbodies are more frequently polluted by organic matter [45] and heavy metals [46]. Non-polluted waterbodies were observed to present Chironominae, Orthocladiinae, and Tanypodinae, while the presence of some Chironominae species indicated pollution [47]. The high abundance of Chironominae species in our study indicates a possible presence of pollution in Jinyang Wetland Park waterbodies.

Regarding the seasonal dynamics of non-biting midges, summer and autumn sites presented higher diversity than spring sites. The species composition varied across the three seasons, and the variation was significantly correlated with environmental conditions such as the barometric pressure, temperature, relative humidity, and wind speed. The diversity of adult non-biting midges emerging from waterbodies is associated with the immature aquatic species. Seasonal variation has been observed in immature wetland species with differing water regimes and nutrient statuses [15]. Summer sites and autumn sites shared the highest similarity in the immature species, and winter sites presented the lowest diversity [11]. Adult non-biting midges have a short lifespan. Thus, the composition of adult species largely depends on the diversity of the immature species. Given the low diversity of the immature species in winter and the subsequent spring, the adults emerging in spring exhibit correspondingly low diversity. After emerging from waterbodies, non-biting midges live a terrestrial life. Their behaviors are guided by endogenous genetics [48] and influenced by environmental factors. We took the barometric pressure, temperature, relative humidity, and wind speed into consideration because they are the factors that affect flying adults. These factors are involved in modulating various activities in insects. Low barometric pressure extended the flight distance and flight duration of flying insects [49]. The reduction in barometric pressure from spring to summer was conducive to attracting non-biting midges from more distant locations. Flying insect biomass increased linearly with temperature across Germany [50]. It is known that the flight duration increases with increases in temperature [51], and suitable temperatures were also found to be conducive to the flight initiation of insects [52]. The increase in temperature from spring to summer facilitated the flight initiation of non-biting midges, thereby enhancing the observed species diversity. An increase in relative humidity could improve the success rate of adult emergence [53]. The seasonal increase in non-biting midge diversity was partly attributed to the improvement in emergence success rate. The crepuscular flight activity of walnut twig beetle *Pityophthorus juglandis* had a negative exponential relationship with increasing wind speed [54]. The flight activity of non-biting midges also declined with wind speed [55]. In our study, compared to autumn, summer had higher wind speeds and lower non-biting midge diversity. Autumn and spring had similar wind speeds. The differences in non-biting midge diversity might be attributed to other factors. In conclusion, our results support significant relationships between non-biting midge community variations and environmental factors. The results extend our knowledge about the activity patterns of non-biting midges and provide a research foundation for developing management strategies for controlling this insect assemblage. It should also be noted that the constrained factors accounted for no more than 27.41% of inertia. The distance to the waterbody, the density of vegetative cover, the duration of the day, and other factors were not included in this work. When observing the population dynamics of non-biting midges, we should take more factors into consideration to estimate their impacts on the diversity of non-biting midges.

## Figures and Tables

**Table 1:** Code and environmental conditions at each sampling event.

Code	Season	Sampling Site	Barometric Pressure (hPa)	Temperature (°C)	Relative Humidity (%)	Wind Speed (m/s)
AutS1R1	Autumn	Site 1	1010.72	18.28	70.26	4.29
AutS1R2	Autumn	Site 1	1014.36	20.94	71.34	1.29
AutS1R3	Autumn	Site 1	1013.29	22.71	72.17	3.40
AutS2R1	Autumn	Site 2	1014.12	22.10	70.92	3.72
AutS2R2	Autumn	Site 2	1011.04	19.10	72.08	2.11
AutS2R3	Autumn	Site 2	1012.27	18.60	70.36	2.67
AutS3R1	Autumn	Site 3	1013.54	18.36	70.56	1.45
AutS3R2	Autumn	Site 3	1014.17	21.95	71.73	2.28
AutS3R3	Autumn	Site 3	1013.11	20.57	70.40	2.04
SprS1R1	Spring	Site 1	1012.79	18.40	78.43	2.79
SprS1R2	Spring	Site 1	1013.19	17.64	77.66	1.41
SprS1R3	Spring	Site 1	1009.73	15.88	75.72	2.81
SprS2R1	Spring	Site 2	1011.52	16.44	78.21	2.71
SprS2R2	Spring	Site 2	1012.96	16.85	77.29	1.71
SprS2R3	Spring	Site 2	1014.01	16.65	78.58	2.30
SprS3R1	Spring	Site 3	1010.87	16.51	77.98	2.44
SprS3R2	Spring	Site 3	1010.86	17.42	77.56	2.69
SprS3R3	Spring	Site 3	1011.03	17.70	74.73	1.70
SumS1R1	Summer	Site 1	1004.28	26.15	89.64	2.80
SumS1R2	Summer	Site 1	1003.30	29.99	79.06	2.97
SumS1R3	Summer	Site 1	999.21	32.13	74.06	4.35
SumS2R1	Summer	Site 2	1003.78	30.83	92.62	3.09
SumS2R2	Summer	Site 2	998.05	28.28	85.63	4.15
SumS2R3	Summer	Site 2	1003.91	27.96	75.53	3.67
SumS3R1	Summer	Site 3	1000.72	30.72	83.46	4.76
SumS3R2	Summer	Site 3	1002.28	29.91	81.54	3.10
SumS3R3	Summer	Site 3	1003.01	29.48	88.45	3.59

**Table 2:** Species identification results based on *COI* sequences.

Subfamily	Tribe	Species	Search Database	Top Similarity (%)
Chironominae	Chironomini	*Benthalia carbonaria*	BOLD	99.23
		*Chironomus agilis*	NCBI	99.37
		*Chironomus circumdatus*	BOLD	99.65–100
		*Chironomus claggi*	NCBI	99.56
		*Chironomus flaviplumus*	BOLD	99.48–100
		*Chironomus fujitertius*	BOLD	98.62–98.79
		*Chironomus javanus*	BOLD	99.82–100
		*Chironomus kiiensis*	BOLD	99.65–100
		*Chironomus nippodorsalis*	BOLD	99.85
		*Chironomus striatipennis*	BOLD	99.65–100
		*Dicrotendipes nervosus*	BOLD	99.65–100
		*Dicrotendipes pelochloris*	BOLD	99.14–100
		*Endochironomus pekanus*	BOLD	100
		*Glyptotendipes tokunagai*	BOLD	100
		*Harnischia longispuria*	BOLD	100
		*Harnischia ohmuraensis*	BOLD	99.14–99.66
		*Kiefferulus glauciventris*	BOLD	99.48
		*Kiefferulus tainanus*	BOLD	99.83–100
		*Microchironomus tabarui*	BOLD	99.83–100
		*Microchironomus tener*	BOLD	98.08
		*Parachironomus gracilior*	BOLD	99.31
		*Polypedilum okiharaki*	BOLD	98.62–99.66
		*Polypedilum harteni*	BOLD	99.31
		*Polypedilum johannseni*	BOLD	99.83
		*Polypedilum masudai*	BOLD	99.31
		*Polypedilum nubifer*	BOLD	99.83–100
		*Polypedilum* sp.	BOLD	100
		*Polypedilum tigrinum*	BOLD	99.48–99.83
	Tanytarsini	*Tanytarsus formosanus*	BOLD	100
Orthocladiinae	/	*Cricotopus sylvestris*	BOLD	99.83–100
	/	*Hydrobaenus kondoi*	BOLD	100
	/	*Limnophyes minimus*	BOLD	100
	/	*Limnophyes verpus*	BOLD	99.83
	/	*Parakiefferiella bathophila*	BOLD	99.83
	/	*Propsilocerus akamusi*	BOLD	99.14
	/	*Smittia aterrima*	BOLD	99.83–100
	/	*Smittia leucopogon*	BOLD	100
	/	*Smittia* sp.	BOLD	100
Tanypodinae	Pentaneurini	*Ablabesmyia monilis*	BOLD	99.66–99.83
	Procladini	*Procladius choreus*	BOLD	100
		*Procladius* sp.	BOLD	99.48
	Tanypodini	*Tanypus chinensis*	BOLD	100

## Data Availability

The *COI* sequences obtained in this study are openly available in GenBank of NCBI at https://www.ncbi.nlm.nih.gov/ (accessed on 20 October 2024) under accession numbers PQ340971–PQ341093.

## Supplementary Materials

The following supporting information can be downloaded at https://www.mdpi.com/article/10.3390/insects15120921/s1: Table S1: NCBI accession numbers of *COI* genes obtained in this study; Table S2: Number of specimens per species at each sampling.
